# Pediatric CIDP: Diagnosis and Management. A Single-Center Experience

**DOI:** 10.3389/fneur.2021.667378

**Published:** 2021-07-02

**Authors:** Małgorzata Łukawska, Anna Potulska-Chromik, Marta Lipowska, Dorota Hoffman-Zacharska, Beata Olchowik, Magdalena Figlerowicz, Karolina Kanabus, Edyta Rosiak, Anna Kostera-Pruszczyk

**Affiliations:** ^1^Department of Neurology, Medical University of Warsaw, Warsaw, Poland; ^2^Department of Medical Genetics, Institute of Mother and Child, Warsaw, Poland; ^3^Department of Child Neurology and Rehabilitation, Medical University of Białystok, Białystok, Poland; ^4^Department of Infectious Diseases and Child Neurology, Poznan University of Medical Sciences, Poznań, Poland; ^5^2nd Department of Radiology, Medical University of Warsaw, Warsaw, Poland

**Keywords:** chronic inflammatory demyelinating polyneuropathy, childhood CIDP, IVIg, atypical CIDP, CIDP criteria

## Abstract

**Background:** Chronic inflammatory demyelinating polyneuropathy (CIDP) is a rare acquired polyneuropathy that especially among youngest children should be differentiated with hereditary neuropathies. Even though upon diagnosis treatment options are similar in children and adults, diagnostic challenges are faced in the pediatric population.

**Methods:** We conducted a retrospective analysis of clinical symptoms, nerve conduction study results, modes of treatment, and final outcome in 37 children aged 3.5–17 years with a final diagnosis of CIDP (18 girls, 19 boys). We established three groups of patients based on age at onset of CIDP: 0–4, 4–13, and 13–18 years. Follow-up ranged from 10 to 222 months.

**Results:** In our analysis, 19/37 patients (51.4%) had an atypical presentation: distal variant of CIDP in 12/37 patients (32.4%) and pure motor variant of CIDP in 5/37 patients (13.5%), and one patient had a pure sensory variant (1/37, 2.7%). Furthermore, 3/37 patients (8.1%) had additional concurring symptoms, including involuntary movements of face muscles (1/37, 2.7%) or hand tremor (2/37, 5.4%). During the follow-up, 23/37 patients (62.2%) received intravenous immunoglobulin (IVIg); 22/37 patients (59.5%) received steroids, 6/37 patients (16.2%) received IVIg and steroids, and 12/37 patients (32.4%) received immunosuppressive drugs, mostly azathioprine, but also methotrexate and rituximab. One patient was treated with plasmapheresis. Complete remission was achieved in 19/37 patients (51.4%) with CIDP in its typical form. Remission with residual symptoms or minimal deficit was observed in 4/37 patients (10.8%), whereas 14/37 patients (37.8%) remain on treatment with gradual improvement.

**Conclusion:** Childhood CIDP may occur in its typical form, but even ~50% of children can present as an atypical variant including distal, pure motor, or pure sensory. Most children have a good prognosis; however, many of them may require long-term treatment. This highlights the importance of an early diagnosis and treatment for childhood CIDP.

## Background

Chronic inflammatory demyelinating polyneuropathy (CIDP) is a rare type of neurological disorder in childhood. Most polyneuropathies occurring in children, ~85%, are hereditary (1). The immune-mediated origin, observed in conditions such as CIDP and Guillain–Barré syndrome (GBS), is responsible for ~9% of causes of childhood polyneuropathy (1). The prevalence rate of CIDP in children is 0.22 per 100,000 (2). CIDP symptoms, by definition, progress gradually over a period of more than 8 weeks in most cases, although especially in children, the disease onset might be acute, developing in <4 weeks, or subacute in 4–8 weeks. An acute onset of childhood CIDP may help establish the correct diagnosis of inflammatory neuropathy. However, slow progression, especially in younger children or even infants, can be overlooked and lead to a Charcot–Marie–Tooth (CMT) disease diagnosis. The course of CIDP may be polyphasic—relapsing–remitting in 61% of patients, or monophasic, progressive in 39% (3). The typical presentation includes symmetric proximal and distal weakness and/or sensory dysfunction of limbs with hyporeflexia or areflexia. To be diagnosed with CIDP, apart from the clinical criteria, the patient should meet electrodiagnostic criteria of demyelination (4–6). Recent studies show the role of immunoglobulin G4 (IgG4) nodal and paranodal antibodies, including antibodies against neurofascin (NF155, NF140, and NF186), contactin and contactin-associated protein (CASPR) (7, 8). Although most cases are typical, there may be some misleading symptoms leading to delay in diagnosis and treatment. In this retrospective study, we describe the clinical course, treatment, and outcomes in a group of 37 children with CIDP.

## Patients and Methods

The study was performed under Bioethical Committee approval no. AKBE/245/2018 (Medical University of Warsaw).

We reviewed the medical records of 37 children diagnosed with CIDP in the Department of Neurology, Medical University of Warsaw, between 2001 and 2019. The analysis population consisted of 18 females and 19 males younger than 18 years. Based on potential difficulties to reach a correct diagnosis especially in the younger population, and in order to evaluate any potential diagnostic challenges in the youngest children, patients were divided into groups by age at onset: very early-onset CIDP (<4 years), early-onset CIDP (4–13 years), and young age-onset CIDP (13–18 years). All patients underwent a clinical assessment and nerve conduction study (NCS); a cerebrospinal fluid test was performed in most of them. The NCS was performed using a Keypoint® EMG device (Skovlunde, Denmark). The spinal magnetic resonance imaging (MRI) protocol included standard sequences: short-TI inversion recovery (STIR) and T2-, T1-, and T1-weighted with gadolinium enhancement. The following sequences were included in the brain MRI protocol: T2-, T1-, and T1-weighted with gadolinium enhancement; Turbo inversion recovery magnitude (TIRM); diffusion-weighted imaging (DWI); and susceptibility-weighted imaging (SWI). The MRI studies were done using 1.5 T (Siemens Avanto) and 3 T (GE Signa) scanners. As part of the differential diagnosis, an MLPA test (SALSA Probemix P405; MRC Holland) for the most common genes causing demyelinating CMT disease (*PMP22, MPZ, GJB1*) was performed. Categorical variables were compared using Fisher exact test. Because of skewed distribution of continuous variables, Kruskal–Wallis test was used to compare the groups. All tests were performed at 0.05 significance level. Pairwise comparisons of the groups were performed if *p*-value for overall comparison was < 0.05. Statistical analyses were performed in R statistical software, version 4.0.3 (Vienna, Austria).

## Results

### Clinical Presentation and Laboratory Results

**Figure 1:**
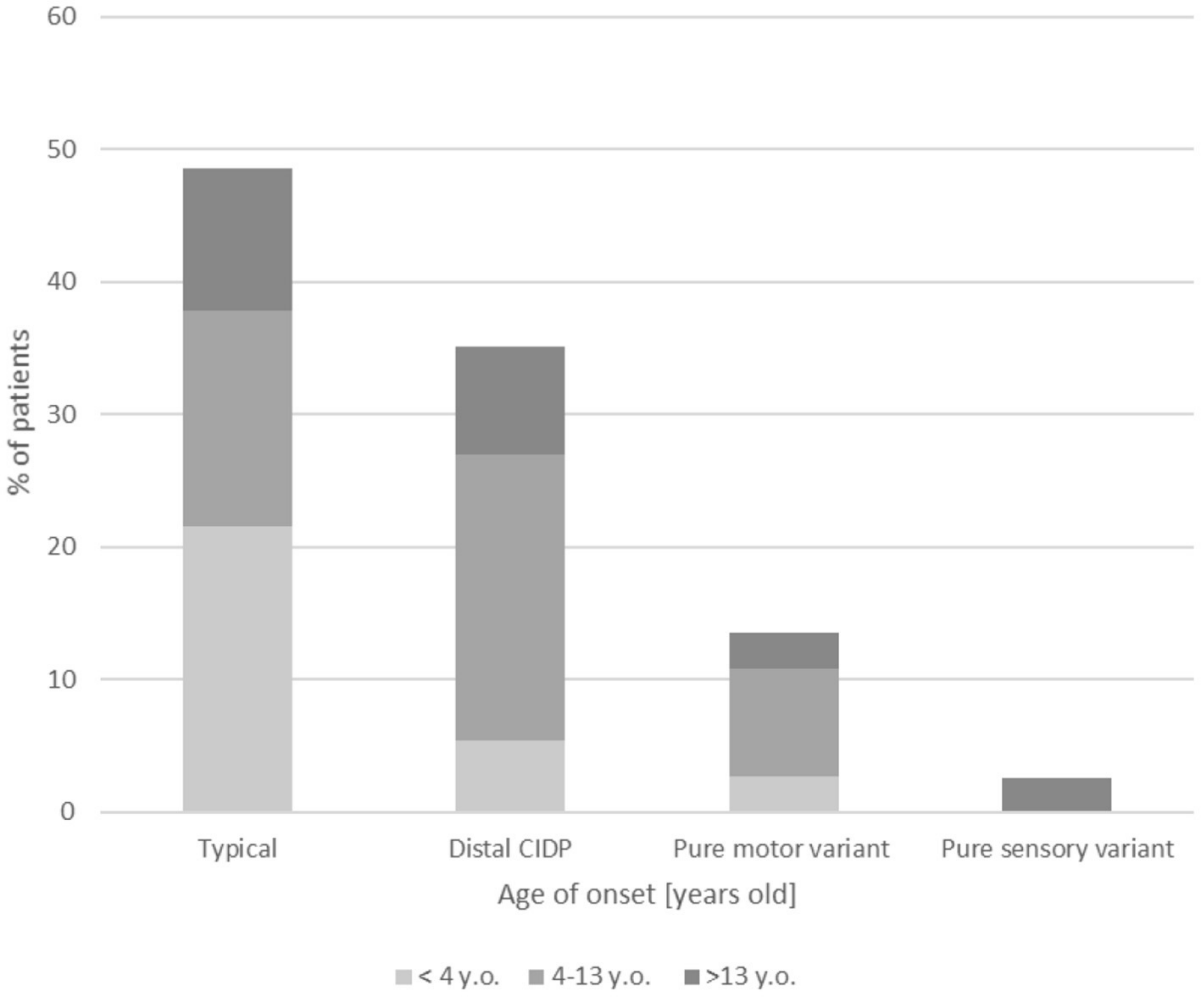
Clinical variants of CIDP among the age groups.

Clinical results are summarized in Table 1. In the differential diagnosis, we have done genetic tests of the most common mutations causing hereditary neuropathies (genes *PMP22, MPZ, GJB1*) in 25/37 patients (67.6%) with all negative results. In most children (21/37, 53.8%), including also patients in the youngest onset age group <4 (8/11, 72.7%), weakness was distributed similarly between proximal and distal weakness (see full summary in Table 1). Cranial nerve dysfunction was observed in 6/37 children (16.2%). Cranial nerve involvement was more frequent in children <4 vs. 4–13 years old (*p* = 0.05) and children 4–13 vs. >13 years old (*p* = 0.032). In our analysis, 19/37 patients (51.4%) had an atypical presentation: distal variant in 13/37 patients (35.1%), pure motor variant in 5/37 patients (13.5%), and pure sensory variant in 1/37 patients (2.6%). Variants of CIDP among our group are shown in Figure 1. Furthermore, 3/37 patients (8.1%) had additional concurrent symptoms, including involuntary facial muscle movements and hand tremor. We have observed autonomic dysfunction in two patients (2/37, 5.4%)—gastrointestinal (constipation) in both. A cerebrospinal fluid examination was performed in most children (28/37, 75.7%) with increased protein >35 mg/dL and normal cytosis <10 cells/mm found in 23/28 (82.1%) of these patients. Among our patients, cytosis in cerebrospinal fluid ranged from 1 to 5 cells/mm (median = 2 cells/mm), whereas protein level 27.5– 871 mg/L (median = 69.5 mg/L). In our group, 11/37 patients were tested for antiganglioside antibodies with eight seropositive cases—details are enclosed in Table 1. None of our patients was checked for other autoantibodies against the Ranvier node–consisting protein.

**Table 1:** Clinical results.

**Clinical features**	**37 children aged 15 months to 17 years [18 girls (48.6%), 19 boys (51.4%)]**	**<4 years 11/37 (29.7%)**	**4–13 years 17/37 (45.9%)**	**>13 years 9/37 (24.3%)**
Type of onset	Acute 4/37 (10.8%)	Acute 0/11	Acute 3/17 (17.6%)	Acute 1/9 (11.1%)
	Subacute 2/37 (5.4%)	1/11 Subacute (9.1%)	Subacute 0/17	Subacute 1/9 (11.1%)
	Chronic 30/37 (81.1%)	10/11 Chronic (90.9%)	Chronic 14/17 (82.4%)	Chronic 6/9 (66.7%)
	Unknown 1/37 (2.7%)	Unknown 0/11	Unknown 0/17	Unknown 1/9 (11.1%)
Preceding event	8/37 (21.6%)	1/11 (9.1%)	4/17(23.5%)	3/9 (33.3%)
	Gastroenteritis 3/37 (7.7%)	Gastroenteritis 1/11 (9.1%)	Gastroenteritis 1/17 (5.9%)	Gastroenteritis 1/9 (11.1%)
	Upper respiratory tract infection 1/37 (2.7%)		Upper respiratory tract infection 1/17 (5.9%)	
	Other: ketoacidosis 1/37 (2.7%), surgery 1/37 (2.7%), neck node enlargement 1/37 (2.7%); hyperglycemia 1/37 (2.7%)		Other: enlargement of lymph nodes on neck 1/17 (5.9%), hyperglycemia 1/37 (2.7%)	Other: surgery for scoliosis 1/9 (11.1%), ketoacidosis 1/9 (11.1%)
Course	Polyphasic 20/37 (54.1%)	Polyphasic 5/11 (45.5%)	Polyphasic 10/17 (58.8%)	Polyphasic 5/9 (55.6%)
	Monophasic 17/37 (45.9%)	Monophasic 6/11 (54.5%)	Monophasic 7/17 (41.2%)	Monophasic 4/9 (44.4%)
Time to diagnosis (months)	1–72 (median = 12)	4–36 (median = 10.5)	1–72 (median = 12)	1–36 (median = 8)
Duration of follow-up (months)	10–222 (median = 55)	24–164 (median = 78)	23–222 (median = 70)	10–183 (median = 37)
Deficit	Deficit motor > sensory 31/37 (83.8%)	Deficit motor > sensory 10/11 (90.9%)	Deficit motor > sensory 14/17 (82.4%)	Deficit motor > sensory 7/9 (77.8%)
	Pure motor 5/37 (13.5%)	Pure motor 1/11 (9.1%)	Pure motor 3/17 (17.6%)	Pure motor 1/9 (11.1%)
	Pure sensory 1/37 (2.7%)			Pure sensory 1/9 (11.1%)
Generalized weakness	21/37 (53.8%)	8/11 (72.7%)	9/17 (52.9%)	4/9 (44.4%)
Proximal weakness	1/37 (2.7%)	1/11 (9.1%)	0/17 (0%)	0/9 (0%)
Cranial nerve involvement	6/37 (16.2%)	3/11 (27.3%): (1) weakness of face muscles (transverse smile + EMG); (2) episodes of choking; (3) ptosis	0/17 (0%)	3/9 (33.3%): (1) anisocoria L>R; (2) fasciculation of tongue muscles; (3) bilateral facial nerve weakness, bilateral damage of trigeminal nerves
Atypical CIDP	18/37 (48.6%)	3/11 (27.3%)	11/17 (64.7%)	4/9 (44.4%)
	Distal CIDP 12/37 (32.4%)	Distal CIDP 2/11 (18.2%)	Distal CIDP 8/17 (47.1%)	Distal CIDP 2/9 (22.2%)
	Pure motor 5/37 (13.5%)	Pure motor 1/11 (9.1%)	Pure motor 3/17 (17.6%)	Pure motor 1/9 (11.1%)
	Pure sensory 1/37 (2.6%)			Pure sensory 1/9 (11.1%)
Additional symptoms	3/37 (8.1%)	1/11 (9.1%)	1/17 (5.9%)	1/8 (11.1%)
	Hand tremor 2/37 (5.4%)		Hand tremor 1/17 (5.9%)	Hand tremor 1/8 (11.1%)
	Involuntary movements of face muscles or head 1/37 (2.7%)	Involuntary movements of oromandibular region—tics 1/11 (9.1%)		
Cerebrospinal fluid dissemination	Performed in 28/37 (75.7%)	Performed in 8/11 (72.7%)	Performed in 11/17 (64.7%)	Performed in 9/9 (100%)
(protein >35 mg/dL, cytosis <10 cells/μL)	Positive 23/28 (82.1%)	Positive 7/11 (63.6%)	Positive 11/17 (64.7%)	Positive 5/9 (55.6%)
MRI nerve root enhancement	Done in 17/37 (45.9%)	Done in 2/11 (18.2%)	Done in 9/17 (52.9%)	Done in 6/9 (66.7%)
	Positive 10/17 (58.8%)	Positive 2/2 (100%)	Positive 6/9 (66.7%)	Positive 2/6 (33.3%)
	Negative 7/17 (41.2%)		Negative 3/9 (33.3%)	Negative 4/6 (66.7%)
Peak modified Rankin Scale (mRS)	4 points; 6/37 (16.2%)	4 points; 3/11 (36.4%)	4 points; 1/17 (5.9%)	4 points; 2/9 (22.2%)
	3 points; 26/37 (70.3%)	3 points; 7/11 (63.6%)	3 points; 15/17 (88.2%)	3 points; 4/9 (44.4%)
	2 points; 4/37 (10.8%)	2 points; 1/11 (9.1%)		2 points; 3/9 (33.3%)
	1 points; 1/37 (2.7%)		1 point; 1/17 (5.9%)	
Follow-up mRS	2 points; 14/37 (37.8%)	2 points; 5/11 (45.5%)	2 points; 7/17 (41.2%)	2 points; 2/9 (22.2%)
	1 point; 4/37 (19.8%)	1 point; 0/11 (0%)	1 point; 2/17 (11.8%)	1 point; 2/9 (22.2%)
	0 points; 21/37 (56.8%)	0 points; 6/11 (54.5%)	0 points; 10/17 (41.2%)	0 points; 5/9 (55.6%)
Antiganglioside antibodies	Done 11/37 (29.7%)	Done 3/11 (27.3%)	Done 4/17 (23.5%)	Done 4/9 (44.4%)
	Positive 8/37 (21.6%)	Positive 3/11 (27.3%)	Positive 2/17 (11.8%)	Positive 3/9 (33.3%)
	Negative 3/37 (8.1%)	Negative 0 /11 (0%)	Negative 2/17 (11.8%)	Negative 1/9 (11.1%)

MRI was done in 17 patients: lumbar spine in nine patients, lumbar spine and contrast-enhanced brain MRI in four patients, and cervical spine MRI in one patient. Three patients had the MRI done outside our center, and we have not had the access to the results, but they were described as normal, without any inflammatory changes. From the whole group, 10/17 patients (58.8%) had inflammatory changes in the MRI. The two patients with CIDP onset at younger than 4 years presented with intradural nerve root (cauda equina) enhancement. In one of them, intradural nerve root thickening and trigeminal nerve enhancement were seen. Four of seven patients with CIDP onset between 7 and 13 years old presented with intradural nerve root enhancement. Two patients in this onset age group presented with intradural nerve root thickening (one with enhancement and one without). Two of five patients with CIDP onset at older than 13 years presented with intradural nerve root enhancement. One of them showed additional intracranial facial nerve enhancement. No intradural nerve root thickening was present in this group. The nerve root enhancement was mild, with some of the nerves spared. All of them had high signal in STIR. The nerve root thickening was mild in intradural segments and marked in foraminal zone with some of the nerve roots spared.

### Electrophysiology

The NCS was performed in all children. The summary of NCS results is presented in Table 2. It should be noted that NCS in children has its limitations due to a poor cooperation in the young population. The Childhood CIDP criteria by Nevo et al. (5) were fulfilled as definite in 26/37 (70.3%) patients, and 7/37 (18.9%) patients met the criteria of possible CIDP. The European Federation of Neurological Societies/Peripheral Nerve Society CIDP electrophysiological definite criteria (2010) (4) were fulfilled in 35/37 patients (94.6%). In 30/37 children (81.1%), we have observed the non-homogeneous pattern of demyelination including difference of 10 m/s in motor conduction velocity between two corresponding nerves (either different nerves from the same limb or the same nerve from different sides) (5), which was present in 30/37 patients (81.1%) and non-homogeneous sensory involvement presented as abnormal median/normal sural sensory nerve action potential (SNAP) (AMNS) (5) seen in 4/37 children (10.8%).

**Table 2:** Summary of electrophysiological results.

**CIDP criteria**	**37 children 15 m.o.−17 y.o. 18 girls (48.6%), 19 boys (51.4%)**	**<4 y.o. 11/37 (29.7%)**	**4–13 y.o. 17/37 (45.9%)**	**>13 y.o. 9/37 (24.3%)**
The Childhood CIDP criteria	Definite 26/37 (70.3%)	Definite 10/11 (90.9%)	Definite 12/17 (70.6%)	Definite 4/9 (44.4%)
Nevo et al., (5)	Possible 7/37 (18.9%)	Possible 1/11 (9.1%)	Possible 3/17 (17.6%)	Possible 3/9 (33.3%)
	Not fulfill 3/37 (8.1%)		Not fulfill 2/17 (11.8%)	Not fulfill 2/9 (22.2%)
EFNS/PNS electrophysiological	Definite 35/37 (94.6%)	Definite 11/11 (100%)	Definite 17/17 (100%)	Definite 7/9 (77.8%)
criteria [2010]	Not fulfill 2/37 (5.4%)			Not fulfill 2/9 (22.2%)

### Disease Course

Predominantly (30/37, 81.1%), the nadir was seen after more than 8 weeks; however, the time of onset, especially in the early-onset and very early-onset groups, was difficult to estimate.

Time from disease onset to diagnosis ranged from 1 to 72 months (median = 12 months). Overall, in both groups of younger children, onset before the age of 4 years and in the group 4–13 years old, 15/28 patients (53.6%) were diagnosed more than 1 year from the onset of the first symptoms, whereas in the group with onset at 13 years or older, only 4/9 children (44.4%) were diagnosed after more than a year.

Acute onset (<4 weeks) was seen in 4/37 patients (10.8%), whereas subacute onsets (4–8 weeks) were observed in 2/37 patients (5.4%). In the onset age group older than 13 years, only one patient presented with acute onset and cranial nerve involvement compared to 3/28 children (10.7%) younger than 13 years who had an acute presentation. A preceding event was reported in 7/37 patients (18.9%), mostly gastroenteritis (3/37, 7.7%) but also upper respiratory tract infection (1/37, 2.7%). The course of the disease was polyphasic with relapses in more than half of the cases (20/37, 54.1%), whereas monophasic course with improvement was observed in 17/37 patients (45.9%).

The highest modified Rankin Scale (mRS) score during the whole observation in most children was 3 (26/37, 70.3%); six patients (16.2%) were unable to walk by themselves. None of the patients scored 5 points in the mRS scale.

The clinical course was comparable between age groups for most variables. Peak mRS was higher in 4–13 vs. >13 years old (*p* = 0.025). For the rest of the variables, differences were not statistically significant; however, the total of 37 patients is too small to reach adequate power of the tests.

### Treatment

Table 3 shows an overview of the treatments received by patients. All children exhibited a complete or partial response to the immunomodulatory therapy. During the entire observation period (between 2001 and 2019), 23/37 children (62.2%) were treated with intravenous immunoglobulin (IVIg) alone compared to 22/37 patients (59.5%) treated with steroids alone. The IVIg was given initially in dose 2 g/kg given during 5 days, the maintenance dose was 0.4–1.2 g/kg every 2–6 weeks. Steroids were given either orally—prednisolone in dose 1 mg/kg per day or intravenous (IV) methylprednisolone in dose 300–500 mg/day during 3–5 days.

**Table 3:** Treatment.

**Treatment**	**37 children 15 m.o.−17 y.o. (18 girls [48.6%], 19 boys [51.4%])**		** <4 y.o. 11/37 (29.7%)**		**4–13 y.o. 17/37 (45.9%)**		**>13 y.o. 9/37 (24.3%)**	
IVIG treatment	23/37 (62.2%)	Full remission 8/37 (21.6%)	6/11 (54.5%)	Full remission 1/11 (9.1%)	9/17 (70.6%)	Full remission 3/17 (17.6%)	8/9 (88.9%)	Full remission 4/9 (44.4%)
		Remission with residual symptoms 4/37 (10.8%)				Remission with residual symptoms 1/17 (5.9%)		Remission with residual symptoms 1/9 (11.1%)
		Still on treatment 4/37 (10.8%)		Still on treatment 2/11 (18.2%)		Still on treatment 1/17 (5.9%)		Still treated 1/9 (11.1%)
		Insufficient response 7/37 (18.9%)		Insufficient response 3/11 (27.3%)		Insufficient response 4/17 (23.5%)		Not sufficient response 7/37 (18.9%)
Steroids treatment	22/37 (59.5%)	Full remission 5/37 (13.5%)	6/11 (54.5%)	Full remission 3/11 (27.3%)	13/17 (76.5%)	Full remission 1/17 (5.9%)	3/9 (33.3%)	Full remission 1/9 (11.1%)
		Remission with residual symptoms 1/37 (2.7%)				Remission with residual symptoms 1/17 (5.9%)		
		Still on treatment 5/37 (13.5%)				Still on treatment 4/17 (23.5%)		Still treated 1/9 (11.1%)
		Insufficient response 11/37 (29.7%)		Insufficient response 3/6 (50%)		Insufficient response 7/17 (41.2%)		Not sufficient response 1/9 (11.1%)
IVIG + steroids treatment	6/37 (16.2%)	Full remission 1/37 (2.7%)	3/11 (27.3%)	Full remission 1/11 (9,1%)	2/17 (11.8%)		1/9 (11.1%)	
		Still on treatment 1/37 (2.7%)		Still on treatment 1/11 (9,1%)				
		Insufficient response 4/37 (10.8%)		Insufficient response 1/11 (9.1%)		Insufficient response 2/17 (11.8%)		Not sufficient response 1/9 (11.1%)
Plasmapheresis	1/37 (2.7%)	Still on treatment 1/37 (2.7%)	1/11 (9.1%)	Insufficient response 1/11 (9.1%)	0/17 (0%)	0/17 (0%)	0/9 (0%)	0/9 (0%)
immunosuppresive treatment added to IVIg or steroids or both	13/37 (35.1%):	full Remission 6/37 (16.2%)	4/11 (36.4%): AZA 2/11 (18.2%) and Mtx 2/11 (18,2%)	Full remission 1/11 (9,1%) (AZA)	6/17 (35,3%) AZA	Full remission 4/17 (23.5%) (AZA)	3/9 (33.3%): AZA 2/9 (22.2%); Rtx 1/9 (11.1%)	Full remission 1/9 (11.1%) (AZA)
		Remission with residual symptoms 1/37 (2.7%)						Remission with residual symptoms 1/9 (11.1%) (Rtx)
		Still on treatment 4/37 10.8%)		Still on treatment 2/11 (18,2%) (1 AZA, 1 Mtx)		Still on treatment 2/17 (11.8%) (AZA)		
		Insufficient response 2/37 (5.4%)		Insufficient response 1/11 (9.1%) (Mtx)				Not sufficient response 1/9 (11.1%) (AZA)

The IVIg treatment was administered in a similar way in all groups and resulted in more remissions or improvement, whereas 3/37 patients (8.1%) with onset age younger than 13 years remain on treatment compared to 1/37 patients (2.7%) 13 years or older at onset. One patient was treated with plasmapheresis. During the entire observation period, 6/37 patients (16.2%) have received treatment with both IVIg + steroids, and 5/37 patients (13.5%) were additionally treated with azathioprine (AZA). All these patients improved after treatment with full remission reported so far in 6/37 children (16.2%). Immunosuppressive drugs, mostly AZA, were given more often to patients with onset age <13 years (6/17, 35.3%) and <4 years (4/11, 36.4%) compared to the >13-year group (2/9, 22.2%). AZA with IVIg or steroids were given to 9/37 patients (24.3%), with full remission in 6/9 patients (66.7%). Other immunosuppressive drugs, methotrexate (MTX) and rituximab (RTX), were started in two patients (2/37, 5.4%) and one patient (1/37, 2.7%), respectively.

### Follow-Up

**Figure 2:**
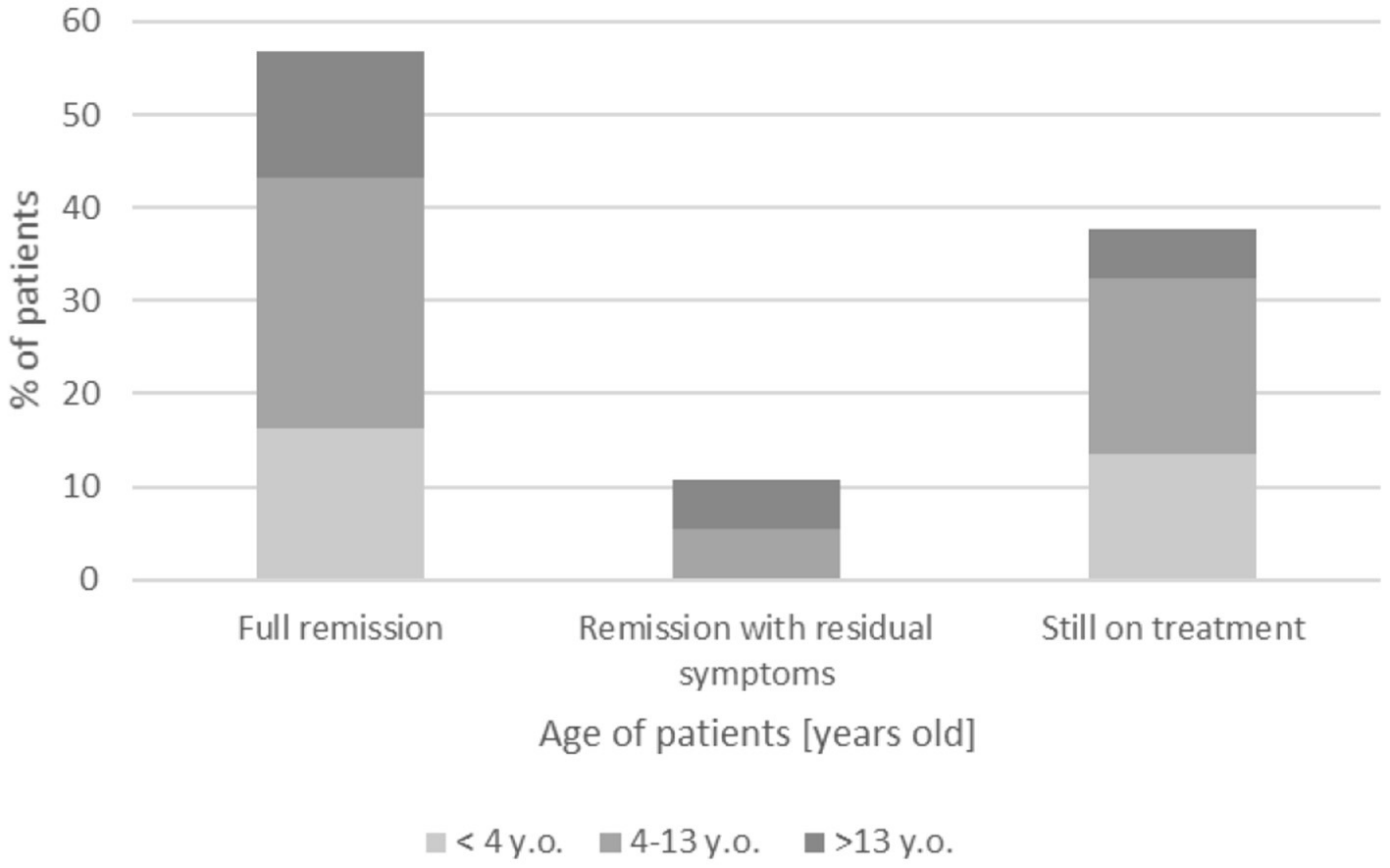
Follow-up of presented patients with CIDP.

The follow-up period ranged from 10 to 222 months (18 years, 8 months); detailed follow-up period is shown in Table 1, whereas the clinical outcome is summarized in Figure 2. A complete remission, defined as no neurological deficit after 1 year with no treatment, was reported in 19/37 children (51.4%) with the typical CIDP form, with similar frequency across all groups: ~50% of patients.

A remission with residual symptoms of minimal deficit was observed in four patients (4/37, 10.8%), whereas 14/37 children (37.8%) continue on treatment with gradual improvement—mostly patients with onset age <13 years (12/14, 85.7%) vs. 2/14 children with onset age >13 years (14.3%).

## Discussion

We present a group of 37 children diagnosed with CIDP at a single center. In patients with onset before the age of 4 years (11/37, 29.7%), the chronic onset of the disease may be overlooked and misdiagnosed as motor delay or CMT. Our subgroups are too small to perform a statistical analysis, but we can observe a trend toward a longer interval between the onset and the final diagnosis in very early-onset and early-onset CIDP.

We excluded from our study children with a suspected genetic, metabolic, or neurodegenerative disorder (including 10 children with initial diagnosis of CIDP and final diagnosis of leukodystrophy, concurring CMT1a, CMTx, and mitochondrial neurogastrointestinal encephalomyopathy syndrome, and mutation of actin α1 gene (ACTA1). Additionally, positive response to treatment and lack of progression of the disease suggest an inflammatory origin. Retrospective data published by Shabo (9) on 118 children with polyneuropathies in order to present an overview of their etiologies showed that 68% of cases had a hereditary etiology.

The prevalence of CIDP in childhood, estimated as 0.23/100,000 to 0.48/100,000 (2, 10), is rarer than in adults: 0.67/100,000 to 1.9/100,000 (2, 10, 11). In our study, no differences between sexes were observed: 18 females and 19 males, similarly to other pediatric CIDP groups in the literature (2, 3, 12), although in adults, more frequent occurrence among males has been described (2).

CIDP is often preceded by an infection, in children this has been described in 23–57% (12–14) vs. 33% in adults (15). In the studied patients, symptoms were preceded and identified by an event in 7/37 patients (8.1%), with similar frequency in patients with onset age of younger than 13 years (4/37, 10.8%) and those older than 13 years (3/37, 8.1%). The most typical triggering factors were infections—gastroenteritis or upper respiratory tract infection—in 4/37 (10.8%), but also acute-onset type 1 diabetes, ketoacidosis, hyperglycemia, surgery for scoliosis, or transient node enlargement. More than 80% (30/37) of patients had an insidious onset of the disease, probably in more than 2 months; more than half of them were referred to our center from other neurological departments after a few months of observation, and for this reason, the correct time of onset and triggering factor could be difficult to estimate.

In contrast to adults, childhood CIDP can more frequently progress for <8 weeks. The subacute form defined as increasing symptoms in 4–8 weeks occurred in 2/37 patients (5.4%) in our analysis group, whereas the acute onset, within <4 weeks, was seen in 4/37 patients (10.8%). The rapid onset was seen in 4/37 patients (10.8%) with onset age younger than 13 years and 2/37 children (5.4%) older than 13 years. A progression of symptoms in <4 weeks needs to be differentiated especially from the GBS. Apart from that, if the patient exhibits deterioration after 8 weeks from onset, or when deterioration occurs three times or more, the diagnosis of CIDP is much more likely (16). Our data show that the course of the disease in very early-onset (<4 years) and early-onset (<13 years) CIDP can be more dynamic; however, in most children, the course is typical for chronic neuropathy. Some data indicated that a longer disease onset predicts also a long-term disability (12, 17, 18). These authors also reported that slow-onset (>3 months) patients required more immunosuppressive treatment and a longer time for recovery than the acute-onset (<3 months) patients. In rapid-onset subgroups, cranial nerve involvement and sensory dysfunction were more common (19).

Even though very early presentation of CIDP before the age of 3 years is rare, it has been reported in the literature. The youngest case of CIDP (20) was a neonate with severe congenital CIDP (hypothesized as a consequence of an expression of fetal myelin antigen and/or antibody transfer between mother and fetus) followed by a complete spontaneous resolution. Congenital CIDP was also suspected in two siblings in the study by Silwal, with later diagnostic revision to GBS (21). He also presented three children with an onset age between 2 and 3 years, and two of them were diagnosed with possible CIDP. Our study indicates 11 cases of CIDP onset at or younger than 4 years of age, with the earliest onset of symptoms at the age of 15 months, preceded by gastroenteritis. Almost all of them (10/11, 90.9%) met the clinical and electrophysiological childhood CIDP criteria by Nevo et al. (5) for definite CIDP.

All but two patients suffered from predominant gait disturbances; in 7/37 children, this symptom was very severe. The most common observed deficit was sensorimotor (32/37, 83.8 %); however, the pure motor variant was found in 5/37 patients (13.5%), and pure sensory involvement was seen in 1/37 patients (2.7%). The literature describes the pure motor variant more often in children groups than in adults, 4–10% (22). In our analysis, only one girl in the onset age group older than 13 years had the pure motor variant, but this variant was present in three patients with onset at age 4–13 years and one patient with onset age at younger than 4 years.

The most typical location for weakness in CIDP, as in the clinical criteria proposed by Nevo et al. (5), is generalized symmetric or proximal, but the distal variant is more common in children, 70% (17). In our analysis, 13/37 patients (35.1%) had distal weakness. Additional symptoms, including hand tremor and involuntary movements as described in the literature (14), were also observed in our analysis in 3/37 children (8.1%). Two patients (5.4%) exhibited hand tremor (one patient with onset age 4–13 years and one from the onset age group >13 years), and one patient with onset age at younger than 4 years had involuntary movements of the face and oromandibular region.

Previous reports by Silwal et al. (21), Costello et al. (23), and Riekhoff et al. (24) indicate cranial nerve involvement in childhood CIDP. Data from six children (16.2%) (Table 1) in our analysis confirm a possible involvement of third, trigeminal, facial, vagal (bulbar), and sublingual nerves accompanied by chronic polyneuropathy. Involvement of cranial nerves is not typical for GBS only, physicians should be aware of its presence also in CIDP. Moreover, some authors highlight that eye signs, cranial nerve palsies, and bulbar disturbances in children with CIDP may be the only presenting symptom (24).

In our study, only two patients (5.4%) manifested with autonomic dysfunction; however, the retrospective character of our study may influence the small frequency of these symptoms. Dysautonomia is described in different studies on adults with CIDP with variable prevalence from 21 to 76% (25). In groups of children with CIDP, autonomic symptoms are not always described; however, in the study by Cabasson et al. (19) in a group of 31 patients, 20% of them have autonomic dysfunction.

Although clinically CIDP is described by symmetrical symptoms, the changes in NCS are more often asymmetric—in our group, in 30/37 patients (81.1%). This non-homogeneous pattern is observed in parameters such as difference of 10 m/s in motor conduction velocity between two corresponding nerves and non-homogeneous sensory involvement presented as AMNS. The mentioned parameters are included in the Childhood CIDP criteria as one of the supportive criteria by Nevo et al. (5) and are helpful in distinguishing the inflammatory origin of neuropathy from hereditary (26).

As previous studies have shown, NCS results, including possible axonal changes (no or very low amplitude), do not reflect neither the disease severity nor response to treatment (27, 28). Features of the inflammation of nerve roots seen in MRI are observed more often in adults, ~60%, than in children, 38% (3). In our analysis, most children did not undergo this examination because of lack of a cooperation or evident electrophysiological features, but the percentage of inflammation was higher than that described in the literature, 10/17 (58.8%).

Latest studies analyze the role of immunoglobulin G4 autoantibodies against nodal and paranodal proteins (NF155, NF140, NF186, contactin, CASPR) in CIDP pathogenesis (7, 8). Patients with these autoantibodies have more specific clinical presentation with possible sensory ataxia, tremor, and poor response to IVIg treatment. However, there may be significant improvement after treatment with RTX, a monoclonal anti-CD20 antibody that eliminates B cells (8). Most of the studies focus on adults, but there have also been reviews in children groups; De Simoni et al. (29) presented 5 seropositive patients among the group of 12 children (41.7%). In our group, we did not find any patient with changes suggestive of the aforementioned characteristics for these autoantibodies' presence.

The discussion about the best first-line treatment in childhood CIDP has been ongoing for many years (30). All treatment options have both proven effectiveness and disadvantages. In general, our findings are in agreement with those of other authors (31). The choice of IVIg as an initial therapy should be made especially among patients with acute or subacute onset or with cranial nerve involvement (similar clinical picture to GBS).

Based on data from the adult study, we chose preferably pulse IV methylprednisolone than daily oral prednisone due to the lower risk of adverse events (AEs) including mainly weight gain and cushingoid features.

Although IVIg usually resulted in a favorable response in 50–88% (5, 14, 19) more often, even in up to 80%, there is a risk of treatment dependency on IVIg as observed in the adult group with CIDP (32). In contrast, steroids were successfully withdrawn in 83%.

Additionally, IVIg and steroids differ with a quicker response to IVIg. The combined treatment with IVIg and steroids has shown good results both in the literature (33) and in our analysis. The proposed mechanism for this phenomenon is that IVIg suppresses the proinflammatory response causing the glucocorticoid resistance, therefore unblocking the effect of steroids (33).

Although some authors suggest the use of non-steroidal immunosuppressive agents in CIDP as controversial (18) and not proven enough to be efficacious (34–37), this treatment needs to be used in some therapy-resistant patients. Our experience indicated that AZA, MTX, and RTX could offer a chance for improvement in patients with persistent limb weakness previously treated with IVIG and steroids. Only a few studies regarded treatment of refractory CIDP. The immunosuppressive or biological therapy is usually added to or follows a previous conventional treatment. Kim et al. (38) reported 10 children refractory to the first-line treatment. In their monophasic group (*n* = 6), four patients were especially responsive to plasmapheresis [plasma exchange (PE)] vs. IVIg. In the polyphasic group, half of treatment-refractory patients received cyclosporine, resulting in a successful disease control. The author suggests that an early administration of plasmapheresis in a monophasic course and cyclosporine in a polyphasic course may be effective treatment options for refractory childhood CIDP. In our population, immunomodulatory agents were also added to maintain a longer remission and allow reducing the dosage of steroids (due to AEs) or IVIg (in case of IVIg shortages in the pharmacy market). The patient treated with RTX exhibited a great rapid improvement (mRS 4 reduced to 1); treatment with MTX also results in a progressive recovery. One patient during the entire observation period was additionally treated with PE (1/37, 2.7%); this patient deteriorated after 3 courses: he experienced a progression of global weakness, anisocoria, and bilateral ptosis. The PE treatment was interrupted, and IVIg infusions were performed with slow recovery and stabilization. No other significant AEs following AZA or MTX were observed. Our data are in accordance with the study by Kim et al. (38); although limited by the small size of the group, they provide a new option for treatment-resistant patients, including the administration of AZA, MTX, RTX, cyclosporine, and plasmapheresis to refractory patients. Thus, in order to implement these treatments, early referral to experienced neuromuscular centers may be crucial for the choice of an appropriate intervention to prevent irreversible axonal damage.

An optimal treatment for CIDP became also a leading question during the COVID-19 pandemic. Fifteen of our patients (15/37, 40.5%) are still on treatment, and we need to consider the best and safest treatment option for our CIDP-affected children. We maintained the IVIg therapy for the new and previously treated IVIg patients in our department. IVIg treatment is not expected to increase the risk of COVID-19 or a severe disease (39). Those patients who are on a corticosteroid therapy should be treated with the lowest possible effective dose, in case of infection, and an additional stress dose should be considered. Therapy cannot be stopped abruptly for children on a chronic treatment.

Based on our results, childhood CIDP was confirmed to be similar to adulthood CIDP; however, it could be a more dynamic disorder with atypical symptoms due to the development of the autoimmune system. CIDP may occur in its typical form, but even ~50% of children can present an atypical variant including distal, pure motor, or sensory. Moreover, the symptoms of CIDP are similar to those of CMT and can lead to a misdiagnosis, especially in very young patients. Our data indicate that the prognosis of pediatric CIDP is good, with residual, mostly minor symptoms or a complete remission in the majority of patients as also observed in literature (31). Our observation of patients with a stable disability (demyelination coexisting with axonal changes) following the first improvement after treatment highlights the importance of an early diagnosis and correct treatment for CIDP.

### Study Limitations

Because of the fact that the study is retrospective, there was limitation of availability to some additional tests, and none of our patients were tested for autoantibodies against the Ranvier node–consisting protein. We also did not perform a nerve ultrasound in any of our patients.

## Data Availability Statement

The raw data supporting the conclusions of this article will be made available by the authors, without undue reservation.

## Ethics Statement

The studies involving human participants were reviewed and approved by Bioethical Commission, Medical University of Warsaw, Warsaw, Poland. Written informed consent to participate in this study was provided by the participants' legal guardian/next of kin. Written informed consent was obtained from the minor(s)' legal guardian/next of kin for the publication of any potentially identifiable images or data included in this article.

## Author Contributions

AK-P and AP-C: idea. AK-P, AP-C, and MŁ: physical examination and data analysis and reasearch. AP-C and MŁ: writting the first version of the manuscript. DH-Z and KK: genetic exam. ML and AP-C: electrophysiological exam. MF and BO: physical examination and data analysis of children from other sites. AP-C, ML, DH-Z, BO, MF, ER and KK: correction. AK-P: final correction. ER: radiological evaluation. All authors contributed to the article and approved the submitted version.

## Conflict of Interest

The authors declare that the research was conducted in the absence of any commercial or financial relationships that could be construed as a potential conflict of interest.
