# Corrigendum: Pediatric CIDP: Diagnosis and Management. A Single-Center Experience

**DOI:** 10.3389/fneur.2021.784144

**Published:** 2022-01-21

**Authors:** Małgorzata Łukawska, Anna Potulska-Chromik, Marta Lipowska, Dorota Hoffman-Zacharska, Beata Olchowik, Magdalena Figlerowicz, Karolina Kanabus, Edyta Rosiak, Anna Kostera-Pruszczyk

**Affiliations:** ^1^Department of Neurology, Medical University of Warsaw, Warsaw, Poland; ^2^Department of Medical Genetics, Institute of Mother and Child, Warsaw, Poland; ^3^Department of Child Neurology and Rehabilitation, Medical University of Białystok, Białystok, Poland; ^4^Department of Infectious Diseases and Child Neurology, Poznan University of Medical Sciences, Poznań, Poland; ^5^2nd Department of Radiology, Medical University of Warsaw, Warsaw, Poland

**Keywords:** chronic inflammatory demyelinating polyneuropathy, childhood CIDP, IVIg, atypical CIDP, CIDP criteria

In the published article, there was an error in the order of the authors in the author list. Instead of “Anna Kostera-Pruszczyk, Anna Potulska-Chromik, Małgorzata Łukawska, Marta Lipowska, Dorota Hoffman-Zacharska, Beata Olchowik, Magdalena Figlerowicz, Karolina Kanabus and Edyta Rosiak,” it should be “Małgorzata Łukawska, Anna Potulska-Chromik, Marta Lipowska, Dorota Hoffman-Zacharska, Beata Olchowik, Magdalena Figlerowicz, Karolina Kanabus, Edyta Rosiak and Anna Kostera-Pruszczyk.”

The authors apologize for this error and state that this does not change the scientific conclusions of the article in any way. The original article has been updated.

## Publisher's Note

All claims expressed in this article are solely those of the authors and do not necessarily represent those of their affiliated organizations, or those of the publisher, the editors and the reviewers. Any product that may be evaluated in this article, or claim that may be made by its manufacturer, is not guaranteed or endorsed by the publisher.

